# Inhibition of NLRP3 Inflammasome: A Prospective Target for the Treatment of Ischemic Stroke

**DOI:** 10.3389/fncel.2020.00155

**Published:** 2020-06-03

**Authors:** Ya-Shuo Feng, Zi-Xuan Tan, Man-Man Wang, Ying Xing, Fang Dong, Feng Zhang

**Affiliations:** ^1^Department of Rehabilitation Medicine, The Third Hospital of Hebei Medical University, Shijiazhuang, China; ^2^Department of Clinical Laboratory Medicine, The Third Hospital of Hebei Medical University, Shijiazhuang, China; ^3^Hebei Provincial Orthopedic Biomechanics Key Laboratory, The Third Hospital of Hebei Medical University, Shijiazhuang, China

**Keywords:** NLRP3 inflammasome, ischemic stroke, inflammatory reaction, death—associated protein kinase, reactive oxygen species

## Abstract

Stroke is one of the major devastating diseases with no effective medical therapeutics. Because of the high rate of disability and mortality among stroke patients, new treatments are urgently required to decrease brain damage following a stroke. In recent years, the inflammasome is a novel breakthrough point that plays an important role in the stroke, and the inhibition of inflammasome may be an effective method for stroke treatment. Briefly, inflammasome is a multi-protein complex that causes activation of caspase-1 and subsequent production of pro-inflammatory factors including interleukin (IL)-18 and IL-1β. Among them, the NLRP3 inflammasome is the most typical inflammasome, which can detect cell damage and mediate inflammatory response to tissue damage in ischemic stroke. The NLRP3 inflammasome has become a key mediator of post-ischemic inflammation, leading to a cascade of inflammatory reactions and cell death eventually. Thus, NLRP3 inflammasome is an ideal therapeutic target due to its important role in the inflammatory response after ischemic stroke. In this mini review article, we will summarize the structure, assembly, and regulation of NLRP3 inflammasome, the role of NLRP3 inflammasome in ischemic stroke, and several treatments targeting NLRP3 inflammasome in ischemic stroke. The further understanding of the mechanism of NLRP3 inflammasome in patients with ischemic stroke will provide novel targets for the treatment of cerebral ischemic stroke patients.

## Introduction

Nowadays, stroke is a major reason for long-term disability and death worldwide, which can lead to a heavy burden on patients and the whole society, especially in low- and middle-income countries (Feigin et al., 2017; Lapchak and Zhang, 2017). It is estimated that one in four adults will experience a stroke in the course of life, and there are at least 80 million stroke survivors worldwide (Feigin et al., 2018; Lindsay et al., 2019). Stroke can cause immediate neurological dysfunction, and even in severe patients, the resultant mass effect and cerebral edema can lead to cerebral herniation and death (Shi et al., 2019). According to a report, death caused by stroke accounted for 11.8% of all deaths in 2015, which is the second leading cause of death in the world next to heart disease (Benjamin et al., 2018). There are many risk factors related to the occurrence of stroke. Recent gene studies on single-gene disorders have shown that common variants at about 35 genetic loci are associated with stroke risk (Dichgans et al., 2019). Besides, a variety of environmental risk factors for stroke have been reported, such as hypertension, smoking, high body mass index (BMI), atrial fibrillation, diabetes, history of stroke and high cholesterol (Donnan et al., 2008; Lu Y. et al., 2014; Hägg et al., 2015). Among these risk factors, hypertension is one of the most leading causes of stroke, accounting for 35% of all strokes (O’Donnell et al., 2010). Furthermore, more than 90% of the stroke burden is attributed to modifiable risk factors, and effective control of metabolic and behavioral risk factors can prevent more than three-quarters of the stroke burden worldwide (Feigin et al., 2016).

Clinically, there are two types of stroke: ischemic stroke and hemorrhagic stroke. Ischemic stroke caused by cerebral artery embolization or thromboembolism, which usually accounts for about 80% of all strokes, while hemorrhagic stroke caused by rupture of the brain’s blood vessel, which accounts for about 15% to 20% of all stroke cases (Gilgun-Sherki et al., 2002). In this mini review article, we focus on ischemic stroke because the incidence of ischemic stroke is much higher than other types of stroke. Among all types of ischemic stroke, focal ischemic stroke is the most common type, which is caused by a cerebral aortic embolization or thrombotic occlusion (transient or permanent) that results in decreased blood flow to a specific area of the brain (McAuley, 1995; Hata et al., 2000; Fann et al., 2013a). Inadequate blood supply can cause cerebral cells to lose essential glucose and oxygen, disrupting the balance of the intracellular environment and triggering pathophysiological processes such as oxidative stress, excitatory toxicity, apoptosis, inflammation, and cell death (Khoshnam et al., 2017).

Specifically, pathophysiology following ischemic stroke is a series of complicated processes, including acidosis, excitotoxicity, bioenergetic dysfunction, destruction of the blood-brain barrier (BBB), toxicity mediated by reactive oxygen species (ROS), infiltration of leukocytes, cytokine-mediated cytotoxicity, loss of cellular ion homeostasis, and production of arachidonic acid products and activation of complement (Woodruff et al., 2011). These various pathophysiological processes trigger each other to form a positive feedback loop, leading to the death of neuronal cell and brain damage eventually (Siesjo, 1992). Among diverse potential mechanisms of stroke, oxidative stress and inflammation are involved in the pathogenesis of cerebral ischemia-reperfusion (I/R) injury, and appropriate regulation of inflammation may have an important effect on the prevention and treatment of stroke (Ahmad et al., 2014).

The treatment of ischemic stroke is based on the re-establishment of blood flow in the ischemic region (Hong et al., 2019). However, the re-establishment process of blood flow can result in further injury to ischemic tissue *via* infiltration of neutrophils, deregulation of cell ion homeostasis, accumulation of ROS, and subsequent inflammatory response leading to cell death (Minutoli et al., 2016). Nowadays, effective treatments for acute ischemic stroke include intravenous injection of tissue-type plasminogen activator (tPA) and endovascular therapy (Schwamm et al., 2013; Yoshimura et al., 2018). However, tPA treatment has limitations because the treatment window is narrow (Schwamm et al., 2013). Besides, endovascular therapy has been reported to be effective for acute cerebral large-vascular occlusion, but its actual effect is unclear (Yoshimura et al., 2018). Based on the above mentioned issues, it is crucial to find an effective and reliable therapy method for ischemic stroke.

In recent, researchers have recognized a new inflammasome signaling pathway—NOD-like receptor pyrin domain containing 3 (NLRP3) inflammasome that may be a crucial mediator in detecting cell injury and mediating inflammation following stroke (Abulafia et al., 2009; Savage et al., 2012; Gustin et al., 2015). Therefore, treatments aiming at NLRP3 upstream and downstream signaling pathways may provide new strategies for treating stroke (Fann et al., 2013b). Here, we summarize the current understanding regarding the structure, assembly, and regulation of the NLRP3 inflammasome, its potential roles in ischemic stroke, and recent treatments targeting at suppressing NLRP3 inflammasome in stroke.

## NLRP3 Inflammasome: Structure, Assembly, and Regulation

### The Structure of NLRP3 Inflammasome

The body’s first line of defense against a variety of diseases is the innate immune system, which is based on various pattern recognition receptors (PRRs) that sense pathogenic microorganisms and other kinds of exogenous or endogenous pathogens, such as damage-associated molecular patterns (DAMPs) and pathogen-associated molecular patterns (PAMPs; Schroder and Tschopp, 2010; Minutoli et al., 2016). When the innate immune system is activated, inflammatory responses can be initiated by the secretion of chemokines and cytokines, resulting in the expression of co-stimulatory and adhesion molecules that can recruit immune cells and activate adaptive immune responses (Abderrazak et al., 2015). NOD-like receptors (NLRs) are a type of PRRs that are expressed primarily in the cytoplasm and can detect signals of intracellular invaders (Martinon and Tschopp, 2005). There are different types of inflammasome-forming NLRs, including NLRP1, NLRP3, NLRP6, NLRP7, NLRP12, NLRC4, NLRC5, and AIM2 (Pedra et al., 2009). Among them, NLRP3 (also termed as cryopyrin or Nalp3) is the most characteristic and closely related to sterile inflammation, which is coded by the cold-induced auto-inflammatory syndrome-1 (CIAS-1) gene and highly expressed in the immune cells and neural cells (Cassel and Sutterwala, 2010; Liu et al., 2013). As a tripartite protein, NLRP3 includes the central NACHT (also termed NOD) domain, the N-terminal pyrin domain (PYD), and the C-terminal leucine-rich repeat (LRR) domain (Franchi et al., 2009b). The LRR domain is involved in mediating autoinhibition and putative ligands, while the NACHT domain is associated with the assembly of inflammasome and self-oligomerization (Duncan et al., 2007; Lamkanfi and Dixit, 2009).

As intracellular oligomeric multiprotein complexes, inflammasomes play an important role in inducing the body’s innate immune response to microbial and injury-related signals (Franchi et al., 2009a). The inflammasome, including the sensor molecule NLR, the pro-inflammatory caspase (pro-caspase-5, pro-caspase-1, or both), and adaptive proteins, can detect various danger signals in the cell, for instance, bacterial RNA and bacterial flagellin (Martinon et al., 2002; Hong et al., 2019). Among various inflammasomes, NLRP3 inflammasome is the most widely studied and is considered to be closely related to sterile inflammation, which is mainly distributed in the cytosol (Tschopp and Schroder, 2010; Li et al., 2018). There are three sections in the NLRP3 inflammasome: the NLRP3 protein, the inflammatory caspase-1, and the adapter protein ASC [Apoptosis-associated Speck-like protein containing a caspase activation recruitment domain (CARD); Abderrazak et al., 2015]. Full-length caspase-1 includes the central large catalytic domain (p20), the C-terminal small catalytic subunit domain (p10), and the N-terminal CARD (Swanson et al., 2019). Besides, ASC contains two protein interaction domains, including the N-terminal PYD and the C-terminal CARD (Swanson et al., 2019).

### The Assembly of NLRP3 Inflammasome

**Figure 1:**
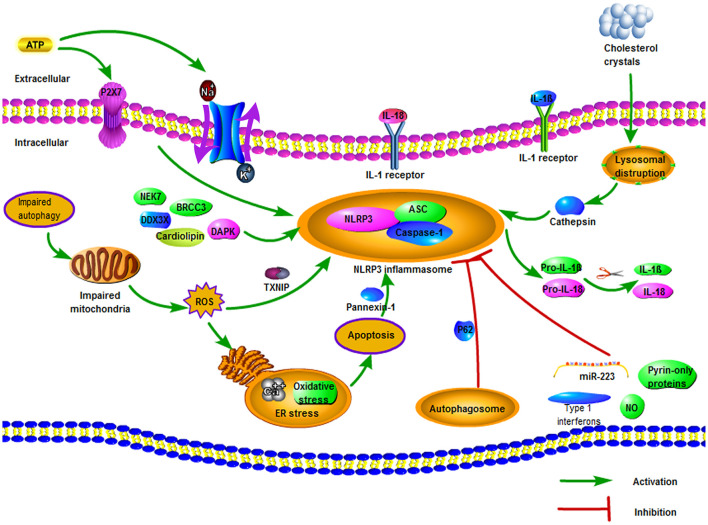
The regulation of NOD-like receptor pyrin domain containing 3 (NLRP3) inflammasome. There are three potential stimuli for NLRP3 inflammasome activation, including reduced intracellular K^+^ concentration, mitochondrial reactive oxygen species (ROS), and lysosomal membrane destruction. Extracellular ATP can promote K^+^ efflux *via* affecting the function of Na^+^/K^+^-ATPase pump and triggering autocrine and paracrine P2X7. Impaired autophagy can increase ROS levels, thereby activating NLRP3 inflammasome and resulting in secretion of Ca^2+^ and oxidative stress. Various particulates, such as the cholesterol crystals of atherosclerotic plaque, can disrupt the lysosomal membrane and deliver cathepsin into the cytoplasm, hence activating the NLRP3 inflammasome. Besides, several negative regulation mechanisms of NLRP3 inflammasome are also demonstrated, including autophagy, nitric oxide (NO), miR-223, type I interferons, and pyrin-only proteins.

As is shown in Figure 1, the activation of the NLRP3 inflammasome is considered as a two-stage process. The first stage, known as the priming stage, is induced by the recognition of PAMPs and DAMPs (Wang et al., 2020). This causes the activation of the NF-κB signaling pathway and promotes the expression of precursor proteins, including the NLRP3, pro-IL-1β, and pro-IL-18 (Shao et al., 2019). The second stage is the activation stage, which is induced by a series of stimulation that occurs during tissue injury, infections, or metabolic imbalances (Zhao and Zhao, 2020). During this stage, stimulation like K^+^ efflux, Na^+^ influx, Ca^2+^ mobilization, chloride efflux, lysosomal damage, ROS, and mitochondrial dysfunction can cause the assembly of the NLRP3 inflammasome (Kelley et al., 2019).

The assembly of NLRP3 inflammasome is initiated by interaction between the pyrin domain of ASC and the pyrin domain of NLRP3 (Vajjhala et al., 2012). After detecting dangerous signals, NLRP3 monomers can be triggered and oligomerized to become definite oligomers (Lechtenberg et al., 2014). These ring structures can recruit ASC monomers to cause the ASC filaments or specks formation by interacting with homophile PYD–PYD (Lu A. et al., 2014). Then, ASC filaments/specks recruit cysteine proteases pro-caspase-1 for assembling inflammasome complexes through the interaction with CARD (Proell et al., 2013). And then, pro-caspase-1 autocatalyzes itself *via* cleavage and activation into active caspase-1, resulting in the subsequent processing of pro-IL-18 and pro-IL-1β into active IL-18 and IL-1β (Ozaki et al., 2015). Furthermore, activated caspase-1 can dissociate gasdermin D (GSDMD) to release its N-terminal domain (Shi et al., 2020). The N-terminal domain of GSDMD binds to phosphatidylserine and phosphatidylinositol phosphates in the cytomembrane to form a 1,020 nm pore and triggers a lytic form of cell death, called pyroptosis (Shi et al., 2015). Pyroptosis is characterized by cytosolic swelling and early rupture of the plasma membrane, which can release DAMPs to trigger inflammatory action, playing major roles in numerous types of immune diseases (Lamkanfi and Dixit, 2010).

In addition to the canonical pathway of the NLRP3 inflammasome in activation of caspase-1, there is also the non-canonical active manner of NLRP3 activated caspase-11 in mice (or the homologs caspase-5 and caspase-4 in humans; Viganò et al., 2015; Yi, 2018). In the non-canonical pathway, caspase-11 may produce abnormal protein secretion and pyroptosis in a manner independent of caspase-1 (Tan et al., 2013). Specifically, caspase-11 can directly recognize and bind to intracellular lipopolysaccharide, leading to its oligomerization and subsequent processing of activation by autoproteolytic cleavage (Kayagaki et al., 2015). And then, caspase-11 can directly cause the cleavage of GSDMD to trigger pyroptosis (Kayagaki et al., 2015; Shi et al., 2015).

### The Regulation of NLRP3 Inflammasome

#### Positive Regulation of NLRP3 Inflammasome

So far, the exact mechanism and cellular stimulation resulting in the activation of NLRP3 inflammasome are not clear (Gao et al., 2017). Py et al. (2013) suggested that the deubiquitination mechanism plays an important role in regulating the NLRP3 inflammasome activation and BRCC3 is a key regulator of NLRP3 activity *via* promoting deubiquitination. Death-associated protein kinase (DAPK) is considered as a crucial molecule, which is necessary for a full generation of IL-1β and accurate assembly of NLRP3 inflammasome (Chuang et al., 2011). Recently, it has been reported that stress granule protein DDX3X can interact with NLRP3 to activate inflammasome, and assembly of stress granule could suppress the activation of NLRP3 inflammasome *via* the sequestration of DDX3× (Samir et al., 2019). Besides, Sharif et al. (2019) demonstrated that NIMA-related kinase 7 (NEK7) could mediate the activation of the NLRP3 inflammasome by bridging adjacent NLRP3 subunits with bipartite interactions. Upon inflammasome activation, the interaction between NEK7 and NLRP3 is enhanced to form a complex that is critical to form ASC speck and activate caspase-1 (He et al., 2016; Shi et al., 2016). Although our understanding of NLRP3 inflammasome has gradually increased in recent years, we need more studies to further clarify the detailed mechanism of NLRP3 in the process of stroke in the future.

Different activators of the NLRP3 inflammasome complex have been reported, including exogenous (such as tissue injury, infection, and metabolic imbalance) and endogenous factors (such as Aβ fibrils, hyaluronan, extracellular ATP, and uric acid crystals; Lamkanfi and Dixit, 2009; Koizumi et al., 2012). Because of the large amount of NLRP3 inflammasome activators, it seems unlikely that all of them will bind to the NLRP3 structure to form the NLRP3 inflammasome (Zhou et al., 2016). In general, researchers have reported three potential stimuli for the activation of the NLRP3 receptor: decreased intracellular K^+^ concentration, mitochondrial ROS, and lysosomal membrane destruction (Martinon et al., 2002; Garg, 2011).

##### Decreased Intracellular K^+^ Concentration

In most cases, decreased intracellular K^+^ concentration is an essential upstream event during the activation of NLRP3 inflammasome (Swanson et al., 2019). Extracellular ATP is a typical danger signal to activate NLRP3 inflammasome, which can affect the function of Na^+^/K^+^-ATPase pump, and the increased Na^+^ influx through aquaporin promotes the osmotic movement of water into the intracellular environment, resulting in K^+^ efflux (Mongin, 2007; Li et al., 2018). Besides, the secretion of ATP can also activate autocrine and paracrine P2X7, resulting in the reduction of K^+^ concentration in the cytoplasm, which activates NLRP3 inflammasome (Lamkanfi et al., 2009). Furthermore, the reduction of intracellular K^+^ level could result in the activation of NLRP3 inflammasome through pore-forming bacterial toxins or endogenous ion channels (Pétrilli et al., 2007). Additionally, the K^+^ channel inhibitor glibenclamide effectively inhibited the activation of inflammasome to many NLRP3 activators (Lamkanfi and Dixit, 2009).

##### Mitochondrial ROS

ROS has been shown to exert important effects on the activation of NLRP3 inflammasomes, which was mainly related to the function of mitochondria (Gross et al., 2011). ROS is primarily produced in the mitochondrial inner membrane, which is closely associated with the enzyme complex in the mitochondrial respiratory chain (Liu et al., 2002). Approximately 2% of the oxygen in normal mitochondria can be converted into ROS (Wang S. et al., 2019). Under normal physical conditions, the small amounts of ROS can be removed by several endogenous antioxidant systems (Sanderson et al., 2013). However, when mitochondria are impaired or oxygen supply is insufficient, a large number of ROS can be produced, further aggravating the damage of mitochondrial structure and function (Turrens, 2003). Increased level of ROS can induce oxidative stress and release of Ca^2+^, cause endoplasmic reticulum (ER) stress, cellular organelles injury, and lead to apoptosis eventually (Gao et al., 2017). Autophagy (i.e., mitophagy) can remove the ROS-producing mitochondria to protect cells, but this process may not occur effectively because autophagy-related proteins such as Beclin1 and microtubule-associated protein 1 light chain 3B (LC3B) are depleted in case of cellular stress and ischemic brain injury (Tian et al., 2010; Nakahira et al., 2011). Thus, impaired autophagy promotes the accumulation of impaired mitochondria in the cytoplasm, thereby increasing ROS levels that activate the NLRP3 receptor (Nakahira et al., 2011; Zhou et al., 2011). Also, the thioredoxin-interacting protein (TXNIP), as a ROS-sensitive regulator, could activate NLRP3 inflammasome (Zhou et al., 2010). In unaffected cells, TXNIP is bound to and inhibited by the oxidoreductase thioredoxin (Fann et al., 2013a). With an increased level of cytoplasmic ROS, this complex begins to dissociate and causes the TXNIP to bind to the NLRP3 receptor (mainly in LRR), resulting in activation of NLRP3 receptor (Zhou et al., 2010; Lane et al., 2013).

##### Lysosomal Membrane Destruction

Lysosomal disruption is also one of the well-known mechanisms of NLRP3 inflammasome activation (Li et al., 2018). NLRP3 inflammasome is activated by cathepsin delivered into cytoplasm because of lysosomal membrane damage, which is triggered by crystalline or particulate structure (Gao et al., 2017). Phagocytosis of a variety of particulates, whether self-originated particulates such as cholesterol crystals and uric acid or foreign-originated particulates such as silica, alum, and asbestos, results in lysosomal disruption and delivering the particulates into the cytoplasm (Hornung et al., 2008). For instance, the cholesterol crystals of atherosclerotic plaque in the location of occlusion can be taken by the endosomes and fuse with the lysosomes, resulting in disruption of lysosomal membranes and release of cathepsin into the cytoplasm (Duewell et al., 2010). Besides, Yamasaki et al. (2009) demonstrated that cathepsin B or cathepsin B-modified proteins are necessary for NLRP3 inflammasome activation; and the Cathepsin B inhibitor, CA-074-Me, could partially suppress NLRP3 activation (Dostert et al., 2008; Bruchard et al., 2013). Furthermore, Okada et al. (2014) suggested that the TAK1-JNK pathway was triggered *via* lysosome rupture and that this activation played an important role in the NLRP3 inflammasome formation *via* the ASC oligomerization.

##### Other Mechanisms

There are several other mechanisms involved in the activation of the NLRP3 inflammasome. The ER is the main intracellular organelle for protein synthesis and processing, and the main calcium reservoir for maintaining calcium homeostasis (Bauernfeind et al., 2011; McCaffrey and Braakman, 2016). ER stress can affect the activation of NLRP3 inflammasome through a variety of effects including calcium or lipid metabolism, the unfolded protein response (UPR), and the production of ROS (Chen X. et al., 2019). Recently, Piippo et al. (2018) suggested that oxidative stress strongly promoted the activation of NLRP3 inflammasome upon dysfunctional cellular clearance. Besides, NLRP3 inflammasome can also be activated by Ca^2+^ mobilization regulated mitochondrial injury and dysfunction (Lee et al., 2012; Murakami et al., 2012). Mitochondria related cardiolipin also plays an important role in the recruitment and activation of NLRP3 inflammasome (Iyer et al., 2013). Also, intrinsic and extrinsic apoptosis is contributed to driving the assembly of NLRP3 inflammasome *via* activating pannexin-1 (Chen K. W. et al., 2019).

#### Negative Regulation of NLRP3 Inflammasome

A series of studies demonstrated that many factors can negatively regulate the activity of NLRP3 inflammasome. Autophagy can remove injured mitochondria, prevent mtDNA, and ROS release into the cytoplasm and block assembly of NLRP3 inflammasome (Nakahira et al., 2011). What is more, autophagosome can degrade NLRP3 inflammasome *via* autophagy adaptor p62 (Harris et al., 2011; Shi et al., 2012). Further, Zhou et al. (2011) reported that 3-MA, an autophagy inhibitor, was able to induce the NLRP3 inflammasome activation. Besides, nitric oxide (NO) has effects on suppressing NLRP3 inflammasome activation through the stabilization of mitochondria, in both humans and mice (Mao et al., 2013). Additionally, it has been shown that NLRP3 could be directly regulated by miR-223 because NLRP3 mRNA includes a conserved miR-223 binding region in its 3’UTR (Yang et al., 2015). Other negative regulators also have been reported, such as type I interferons and pyrin-only proteins (Guarda et al., 2011; de Almeida et al., 2015).

## Involvement of the NLRP3 Inflammasome in the Pathophysiological Processes Following Ischemic Stroke

**Figure 2:**
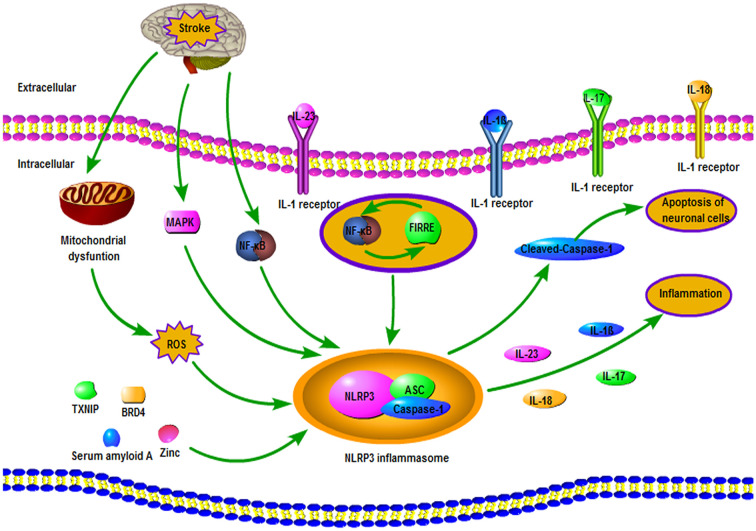
Involvement of the NLRP3 inflammasome in the pathophysiological processes following ischemic stroke. After a stroke, the accumulation of ROS plays an important role in the activation of the NLRP3 inflammasome. Mitogen-activated protein kinase (MAPK) and NF-κB signaling pathways are regarded as crucial mechanisms for regulating NLRP3 inflammasome. Also, a positive feedback loop formed by functional intergenic repeating RNA element (FIRRE) and NF-κB promotes the transcription of NLRP3 inflammasome. Furthermore, NLRP3 inflammasome can regulate apoptosis of neuronal cells *via* cleaved-caspase-1 and promote inflammation by releasing the pro-inflammatory cytokines.

As is shown in Figure 2, several mechanisms can regulate NLRP3 inflammasome after ischemic stroke. Following ischemic stroke, the generation of ROS can activate both cerebral inflammatory reactions and NLRP3 inflammasome, triggering neuronal cell injury, brain edema, and neural dysfunction (Wang et al., 2007; Minutoli et al., 2016). Mitochondrial dysfunction also exerted a crucial role in the activation of NLRP3 inflammasome after OGD/R in microglia, and mitochondrial protector was able to suppress the NLRP3 inflammasome activation in ischemic stroke rats (Gong et al., 2018). Ishrat et al. (2015) report that TXNIP could induce the activation of the NLRP3 inflammasome, resulting in the neuronal damage after ischemic stroke. Besides, serum amyloid A can contribute to the NLRP3 inflammasome activation of microglial cells in ischemic stroke (Yu et al., 2019). Bromodomain-containing protein 4 (BRD4) also plays an important role in the activation of NLRP3 inflammasome in MCAO mice (Zhou et al., 2019). TPEN, a membrane-permeant zinc chelator, can bloke the elevated levels of NLRP3 and caspase-1, suggesting that zinc is closely related to the formation of NLRP3 inflammasome (Park et al., 2020). Furthermore, TPEN inhibits the elevation of NLRP3 inflammasome in the oxygen-glucose deprivation (OGD) model, indicating that increased zinc is essential for the NLRP3 inflammasome activation in OGD models (Park et al., 2020). Besides, mitogen-activated protein kinase (MAPK) and nuclear factor-κB (NF-κB) signaling pathways played an important role in regulating the expression and activation of NLRP3 inflammasomes in brain tissue and primary cortical neurons during ischemia (Fann et al., 2018). Additionally, an *in vitro* research suggested that lncRNA functional intergenic repeating RNA element (FIRRE) and NF-κB could form a positive feedback loop to facilitate NLRP3 inflammasome transcription, hence cause OGD/R injury of microglia in brain (Zang et al., 2018).

Increasing evidence has suggested that NLRP3 inflammasome is a crucial mediator of neuroinflammation and plays an important role in the progression and pathogenesis of ischemic stroke (Fann et al., 2013a). Gong et al. (2018) demonstrated that the NLRP3 inflammasome was firstly activated in microglial cells after brain I/R injury onset and subsequently expressed in microvascular endothelial cells and especially in neurons (Gong et al., 2018). NLRP3 inflammasomes can regulate glial and neuronal cell death in ischemic stroke *via* enhancing generation and secretion of the pro-inflammatory cytokines including IL-1β and IL-18 and through pleiotropic impacts of cleaved-caspase-1 in regulating neuronal cell apoptosis (Fann et al., 2013a). Also, NLRP3 inflammasomes can cause damage to the IL-23/IL-17 axis, thus aggravating cerebral I/R injury (Wang H. et al., 2019).

The expression level changes and abnormal gene mutation of coding components of NLRP3 can impact NLRP3-regulated inflammatory response, thus disturbing the immune balance of the internal environment and the occurrence of ischemic stroke (Fann et al., 2013b; Xie et al., 2014; Yang et al., 2014). For instance, inhibition of NLRP3 significantly prevents neuronal death and reduces I/R injury in both *in vitro* and *in vivo* cerebral ischemic settings (Deroide et al., 2013; Yang et al., 2014). Besides, it was reported that polymorphism of the NLRP3 gene may affect the risk of ischemic stroke *via* changing plaque vulnerability in the Chinese population (Zhu et al., 2016; Cheng et al., 2018). Further, NLRP3 deficiency improved neurovascular damage in mice model following focal ischemic stroke *via* decreasing BBB damage and infarct volumes according to the evaluation of Evans blue permeability, magnetic resonance imaging (MRI), and electron microscopic analyses (Yang et al., 2014).

## Intervention Measures and Related Mechanisms *via* Inhibiting NLRP3 Inflammasome in Ischemic Stroke

### Medicants

Currently, there are a series of molecules that play a neuroprotective role in ischemic stroke models by inhibiting the NLRP3 inflammasome pathway. Lu et al. (2016) suggested that minocycline administrated 1 h following reperfusion improved neurological dysfunction, decreased infarct volume, and reduced cerebral edema *via* inhibiting activation of microglia and NLRP3 inflammasome signaling. IMM-H004, a novel coumarin derivative, could decrease the expression level of chemokine-like factor 1 (CKLF1) combining with C-C chemokine receptor 4, further inhibiting the NLRP3 inflammasome activation and inflammation, thereby exerting therapeutic effects on rats following ischemic stroke (Ai et al., 2019). Additionally, progesterone and steroids 17b-estradiol (P and E2) have positive effects on ischemic stroke. For instance, Lammerding et al. (2016) suggested that P and E2 application following cerebral ischemia decreased the expression of NLRP3, suppressed the inflammatory response, and reduced the infarct volume in transient focal ischemic rat models. Also, Zhang et al. (2017) indicated that Arctigenin pretreatment could reduce the neurological score, infarct volume, and brain water content, by suppressing the level of NLRP3, IL-1β, and IL-18 and activating SIRT1 signaling pathway. Further, these authors administrated EX527 (SIRT1 inhibitor) under oxygen-glucose deprivation (OGD) condition, and they found that EX527 could reverse the suppressive effect of Arctigenin on NLRP3 inflammasome activation, indicating that activation of SIRT1 signaling pathway plays an important role in inhibiting NLRP3 inflammasome activation induced by Arctigenin (Zhang et al., 2017). What is more, it has been reported that pretreatment with umbelliferone for 7 days could improve infarct volume, neurological outcomes, and brain edema in middle cerebral artery occlusion (MCAO) rat models by suppressing TXNIP/NLRP3 inflammasome and activating peroxisome proliferator-activated receptor-γ (PPAR-γ; Wang et al., 2015). Huang et al. (2018) demonstrated that Stachybotrys microspora triphenyl phenol-7 (SMTP-7) could reduce the expression of NLRP3, TNF-α, NF-κB, and cleaved-caspase-3 positive cells in ischemic stroke mice model. Hispidulin could reduce brain edema and infarct size, as well as provide neuroprotective effects *via* inhibiting NLRP3-mediated pyroptosis through regulating the AMPK/GSK3β signaling pathway both *in vitro* and *in vivo* (An et al., 2019). Also, purified anthocyanin extracts (PAEs) could decrease the cerebral infarction volume and brain damage through Toll-like receptor 4 (TLR4) /NF-κB and NLRP3 pathways (Cui et al., 2018). Besides, ruscogenin is a crucial steroid sapogenin derived from *Ophiopogn japonicus*, and ruscogenin could improve neurological dysfunctions of ischemic stroke mice *via* suppressing expression levels of NLRP3, IL-1β, caspase-1, TXNIP, MAPK, and ROS (Cao et al., 2016). Sinomenine, an effective natural anti-inflammatory, and anti-apoptotic molecule inhibited the activity of NLRP3 inflammasome in the cerebral ischemic model, and the protective effect can be reversed by AMPK inhibitors, indicating that suppressive effect of sinomenine on NLRP3 inflammasome was mediated by AMPK pathway (Qiu et al., 2016). In summary, many medicants have positive effects on improving neurological dysfunction, infarct volume, and cerebral edema in ischemic stroke model *via* suppressing NLRP3 pathways.

### Molecular Inhibitors

#### MCC950

MCC950 is an NLRP3-inflammasome inhibitor that has been shown to exert positive effects on ischemic stroke models. Ismael et al. (2018) demonstrated that MCC950 could improve neurological deficits and reduce infarct volumes and edema, which was related to the suppression of cleaved-caspase-1, IL-1β, TNF-α, poly (ADP-ribose) polymerase (PARP) and cleaved-caspase-3 and paralleled less phosphorylated IκBα and NF-κB p65 expressions in ischemic stroke mouse model. Besides, Wang H. et al. (2019) suggested that MCC950 was able to inhibit the expression of IL-23 receptor and the activation of the IL-23/IL-17 axis in ischemic stroke mice model. What is more, it was also reported that MCC950 improved neurological dysfunction at 24 h after cerebral I/R and promoted 28-day survival rate in diabetic mice with ischemic stroke, involving in the mRNA transcription level changes of NLRP3, caspase-1, and IL-1β (Hong et al., 2018). Also, MCC950 exerted beneficial effects on improving the vascular integrity and cognitive dysfunction and preventing the hypoxia-regulated decrease of brain-derived neurotrophic factor (BDNF) secretion in stroke rat models with diabetes (Ward et al., 2019).

#### Other Molecular Inhibitors

Several other molecular inhibitors also have beneficial effects by reducing the expression of NLRP3 in ischemic stroke. NOX inhibitor apocynin and nicotinamide adenine dinucleotide phosphate (NADPH) could inhibit the level of NLRP3, ASC, caspase-1, IL-1β and IL-18 in the cortex, improve the neurological functions, and decrease the infarct volume in ischemic stroke mouse model (Qin et al., 2017). Further, the beneficial effects for the mouse model could be greatly improved by combination therapy of NADPH and NOX inhibitors (Qin et al., 2017). Nafamostat mesilate (NM), as a wide-spectrum serine protease inhibitor, has immune-modulatory impacts on ischemic stroke rats, which is related to the suppression of NLRP3 inflammasome and NF-κB signaling pathway (Li et al., 2016). NM also reduced the level of various pro-inflammatory molecules) including IL-1β, TNF-α, COX-2, and iNOS) and increased the expression of anti-inflammatory factors (including TGF-β, CD206, IL-4, and IL-10; Li et al., 2016). Besides, JQ1, a bromodomain-containing protein 4 inhibitors, has been reported to exert protective effects on ischemic stroke mice *via* several mechanisms, including inhibiting the expression of NLRP3, caspase-1, ASC and gasdermin D, blocking the NF-κB signaling pathway and suppressing glial activation (Zhou et al., 2019). Besides, Bruton’s tyrosine kinase (BTK), as a member of the Tec family structurally associated with spleen tyrosine kinase (Syk), which is related to ASC phosphorylation, can form ASC specks and activate AIM2 and NLRP3 inflammasomes (Hara et al., 2013). Ito et al. (2015) demonstrated that BTK inhibitor (ibrutinib) could effectively influence the activation of the NLRP3 inflammasome, attenuate infarct volume growth and improve the neurological damage, suggesting that BKT is important for the activation of NLRP3 inflammasome. Taken together, all of these molecular inhibitors can inhibit the expression of NLRP3 and have a neuroprotective effect on ischemic stroke. In Table 1, we have summarized several molecular inhibitors mentioned above that can inhibit NLRP3 inflammasome in the cerebral ischemic model.

**Table 1:** Several molecular inhibitors *via* inhibiting NOD-like receptor pyrin domain containing 3 (NLRP3) inflammasome in ischemic stroke.

Treatments	Models	Main effects	Reference
MCC950	Transient MCAO mice model	Inhibiting the level of cleaved-caspase-1, IL-1β, TNF-α, PARP, and cleaved-caspase-3 and paralleled less phosphorylated IκBα and NF-κBp65.	Ismael et al. (2018)
MCC950	Transient MCAO mice model	Suppressing the expression of IL-23 receptor and the activation of IL-23/IL-17.	Wang et al. (2020)
MCC950	Transient MCAO with type 2 diabetic mice model	Inhibiting mRNA transcription levels of NLRP3, caspase-1, and IL-1β.	Hong et al. (2018)
MCC950	Transient MCAO with high-fat diet/streptozotocin-induced (HFD/STZ) diabetic male rats model	Improving vascular integrity and cognitive dysfunction and preventing the decrease of BDNF secretion.	Ward et al. (2019)
Apocynin NADPH	Transient MCAO mice model	Inhibiting the level of NLRP3, ASC, caspase-1, IL-1β, and IL-18 in the cortex.	Qin et al. (2017)
NM	Transient MCAO rats model	Suppressing NLRP3 inflammasome, inflammation, and NF-κB signaling pathway.	Li et al. (2016)
JQ1	Transient MCAO mice model	Inhibiting the expression of NLRP3, caspase-1, ASC, gasdermin D, and the NF-κB signaling pathway.	Zhou et al. (2019)
Ibrutinib	Transient MCAO mice model	Influencing the activation of NLRP3 inflammasome.	Ito et al. (2015)

### Intravenous Immunoglobulin

Intravenous immunoglobulin (IVIg) was used to treat a variety of inflammatory diseases, which could attenuate neuronal cell loss, apoptosis, infarct size, and improve function in ischemic stroke model (Widiapradja et al., 2012). The mechanism by which IVIg protects brain cells from ischemic injury is the inhibition of NLRP3 and NLRP1 inflammasomes, indicating that therapeutic interventions targeting inflammasome assembly and activity have obvious therapeutic benefits (Fann et al., 2013b). Furthermore, Fann et al. (2018) indicated that IVIg induced inhibition of NLRP3 and NLRP1 inflammasomes was mediated by MAPK and NF-κB signaling pathways. Besides, IVIg could also increase the expression of anti-apoptotic proteins, such as Bcl-2 and Bcl-xL (Fann et al., 2018). Therefore, IVIg is a promising therapeutic method for protecting brain cells against cerebral ischemic *via* inhibition of NLRP3 and NLRP1 inflammasomes, which is regulated by the MAPK and NF-κB signaling pathways.

### Electroacupuncture

Electroacupuncture (EA) is a complementary and alternative medical treatment that applies electric currents to specific acupuncture points (Cai et al., 2019). It has been reported that EA treatment could decrease the inflammatory response mediated by NLRP3 inflammasome and regulate the balance between pro-inflammatory and anti-inflammatory cytokines (Jiang et al., 2019). The α-BGT, a 7nAChR antagonist, was able to reverse the EA induced suppressive effects on NLRP3 inflammasome and break the balance between pro-inflammatory and anti-inflammatory factors, suggesting that EA has neuroprotective effects on cerebral ischemic rats by regulating 7nAChR-mediated NLRP3 inflammasome (Jiang et al., 2019). Sha et al. (2019) indicated that EA remarkably reduced the neurological dysfunction and infarct volume, increased level of miR-223 and attenuated expressions of NLRP3, caspase-1, IL-18, and IL-1β. However, these beneficial effects of EA could be partially reversed by antagomiR-223, suggesting that the therapeutic effects of EA are associated with the suppression of the miR-223/NLRP3 pathway (Sha et al., 2019). In a word, EA could decrease the inflammation, infarct volume, and neurological dysfunction through suppressing the expression level of NLRP3 inflammasome mediated by 7nAChR and miR-223 in ischemic stroke.

### Other Therapeutic Methods

Several other therapeutic methods have been used to treat the ischemic stroke animal models by suppressing NLRP3 inflammasome. Intermittent fasting (IF) for 4 months could suppress the inflammation and brain tissue injury in the MCAO mice model *via* suppressing the level of NLRP1, NLRP3, IL-1β, and IL-18, and inhibiting the up-regulation of MAPK and NF-κB signaling pathways (Fann et al., 2014). Human umbilical cord blood mononuclear cells (cbMNCs) transplantation had beneficial effects on improving the neurologic deficits, memory function and learning ability in ischemic stroke rats, which is associated with the activation of NF-κB, inhibition of NLRP3 inflammasome, increased level of vascular endothelial growth factor (VEGF) and Angiopoietin-1, and reduction of cleaved caspase-1 and mature IL-1β (Liu et al., 2018). Besides, light-emitting diode (LED) treatment could decrease neuroinflammatory reactions and brain damage after ischemic stroke *via* reducing cell death, decreasing IL-1β and IL-18, and inhibiting NLRP3 inflammasome, MAPK signaling, TLR-2 levels and NF-κB activation (Lee et al., 2017). Also, ketogenic diets may inhibit ER stress and protect mitochondrial integrity from ischemic brain damage *via* inhibiting mitochondrial transposition of dynamin-related protein 1 (Drp1), thereby suppressing activation of NLRP3 inflammasome and playing a neuroprotective role in ischemic stroke (Guo et al., 2018). Taken together, these above-mentioned treatments could improve neurological dysfunctions following ischemic stroke *via* inhibiting NLRP3 inflammasome.

## Conclusion

In recent years, we have further deepened our understanding of the NLRP3 inflammasome. Increasing evidence has shown that inhibition of NLRP3 may significantly reduce the infarct volume and improve neurological function in cerebral ischemic animal models. In summary, the study of the effect of NLRP3 inflammasome and its potential mechanism in ischemic stroke will provide new therapeutic targets for the treatment of ischemic stroke. On the one hand, the discovery of NLRP3 inflammasome provides a new way to study the molecular mechanism of ischemic stroke. On the other hand, regulation of multiple levels of inflammation targeting NLRP3, such as its assembly, expression, and activation, may provide new ideas for saving penumbral tissue and preventing neurological deterioration after ischemia stroke.

In addition to ischemic stroke, inflammatory pathways also play an important role in the treatment of patients with cardiovascular disease (Grebe et al., 2018; Sardu et al., 2020). Numerous studies demonstrate that the activation of NLRP3 inflammasome plays a crucial role in the occurrence of cardiovascular disorders, such as atherosclerosis, hypertension, myocardial ischemia, cardiomyopathy, infectious cardiac disease, and heart failure (Pasqua et al., 2018; Wang et al., 2018). Furthermore, treatments targeting the NLRP3 inflammasome are effective for the improvement of cardiovascular disease (Wang et al., 2018). Therefore, we emphasize that there may be a correlation on the role of NLRP3 informsome between the cardiovascular and cerebrovascular networks.

However, the exact mechanisms by which NLRP3 inflammasomes perceive various activators are not fully understood and need further research in the future. Also of note, the role of NLRP3 inflammasomes in the pathogenesis of ischemic stroke is not clear and requires further investigation. Furthermore, a key focus of future research is to identify specific NLRP3 inhibitors and related mechanisms *via* our further study on NLRP3 inflammasome activation during a stroke. Besides, due to the complexity of the pathogenesis of ischemic stroke, it is important to determine the stages of disease at which the NLRP3 inflammasome targeted treatment is effective.

## Author Contributions

Y-SF and FZ had the idea for the article. M-MW and Z-XT performed the literature search and data analysis. YX and FD drafted and critically revised the work.

## Conflict of Interest

The authors declare that the research was conducted in the absence of any commercial or financial relationships that could be construed as a potential conflict of interest.
